# Soluble receptor for advanced glycation end-products and progression of airway disease

**DOI:** 10.1186/1471-2466-14-68

**Published:** 2014-04-24

**Authors:** Hiroshi Iwamoto, Jing Gao, Ville Pulkkinen, Tuula Toljamo, Pentti Nieminen, Witold Mazur

**Affiliations:** 1 Heart and Lung Center, Division of Pulmonary medicine, University of Helsinki and Helsinki University Central Hospital, 00014 Helsinki, Finland; 2 Department of Molecular and Internal Medicine, Graduate School of Biomedical Sciences, Hiroshima University, 734-8551 Hiroshima, Japan; 3 Department of Pulmonary Medicine, Lapland Central Hospital, Rovaniemi, Finland; 4 Medical Informatics and Statistics Group, University of Oulu, Oulu, Finland

## Abstract

**Background:**

The receptor for advanced glycation end-products (RAGE) is highly expressed in the lung, where it is believed to have a homeostatic role. Reduced plasma levels of soluble RAGE (sRAGE) have been reported in patients with chronic obstructive pulmonary disease (COPD). The aim of the present study was to evaluate the association of plasma sRAGE levels with a longitudinal decline of lung function. We have also measured plasma levels of high mobility group box 1 (HMGB1), a RAGE ligand which has been associated with chronic inflammatory diseases including COPD.

**Methods:**

Baseline plasma concentrations of sRAGE and HMGB1 were measured in non-smokers (n = 32), smokers without COPD (n = 212), and smokers with COPD (n = 51), and the associations of the plasma sRAGE and HMGB1 levels with longitudinal declines of lung function during a 4-year follow-up period were analysed.

**Results:**

The plasma levels of sRAGE were significantly lower in smokers without COPD and in smokers with COPD, as compared to those of non-smokers. Plasma sRAGE levels positively correlated with FVC and FEV_1_ and inversely correlated with BMI and pack-years. Lower sRAGE levels were associated with greater declines of FEV_1_/FVC over 4 years in all participants. Moreover, multivariate regression analysis indicated that the baseline plasma sRAGE concentration was an independent predictor of FEV_1_/FVC decline in all groups. A subgroup analysis showed that decreased sRAGE levels are significantly associated with a more rapid decline of FEV_1_/FVC in smokers with COPD. There was no significant correlation between plasma HMGB1 levels and longitudinal decline of lung function.

**Conclusions:**

Lower plasma concentrations of sRAGE were associated with greater progression of airflow limitations over time, especially in smokers with COPD, suggesting that RAGE might have a protective role in the lung.

## Background

The receptor for advanced glycation end-products (RAGE) is a cell-surface receptor belonging to the immunoglobulin superfamily [1]. RAGE is a pattern-recognition receptor that binds multiple ligands, and in most normal tissues it is typically expressed at low levels or is undetectable. However, in the lung, RAGE is highly expressed even under normal physiological conditions, and it is believed to have a homeostatic function [2]. We have previously reported that the expression of RAGE is significantly decreased in the COPD lung, especially in severe disease [3]. Recent cross-sectional studies, including one conducted by our group, have consistently shown that circulatory levels of the soluble isoform of RAGE (sRAGE) are reduced in COPD patients [4-8]. Additionally, reduced circulatory sRAGE levels are associated with more severe airflow limitation [8,9], reduced diffusion capacity, and emphysema [6,8] in COPD patients.

High-mobility group box 1 (HMGB1) is a chromatin protein that is released from necrotic cells or activated immune cells [10]. Extracellular HMGB1 is capable of interacting with RAGE or Toll-like receptor and activating a pro-inflammatory cascade [11]. It has recently been shown that HMGB1 is up-regulated in COPD lung tissue and co-localised with RAGE [12]. Furthermore, circulatory HMGB1 levels are elevated in patients with COPD, especially in those with more severe airflow limitation or in cases complicated by comorbid lung cancer [13-15].

The findings of these recent cross-sectional studies have supported an association of RAGE and its ligand HMGB1 with the progression of COPD. However, little is known about the correlation of these molecules and pulmonary function decline over time. The aim of the present study was to perform a longitudinal cohort study to evaluate plasma levels of sRAGE and HMGB1 in non-smokers, smokers without COPD, and smokers with COPD, and to estimate the predictive value of sRAGE and HMGB1 levels for decline of lung function over time. We examined the association between longitudinal changes of spirometric variables during 4 years and baseline plasma levels of sRAGE and HMGB1 along with demographic variables at the baseline visit including age, BMI, smoking status, and spirometric measurements.

## Methods

### Participants

The participants in the present study were part of a longitudinal cohort survey of smokers and non-smokers conducted in northern Finland. The details of the project and the inclusion and exclusion criteria have been published elsewhere [16,17]. In brief, the exclusion criteria were presence of lung disease or other disease; use of regular medication; risk factors for lung disease such as allergies, infections, and exposures; history of asthma or any previous lung infection including pneumonia or bronchiectasis; malignancy; and viral infection during the previous 2 months [16]. Based on a detailed self-reported questionnaire, all participants considered themselves healthy.

All of the smokers in the study had a cigarette smoking history of ≥10 years. The diagnosis of COPD was defined according to the Global Strategy for the diagnosis, management, and prevention of COPD (GOLD) criteria, i.e. FEV_1_/FVC <70% and bronchodilator effect <12% related to long-term smoking [18,19]. All COPD diagnoses in the study cohort were confirmed during the study period; none of the participants had any previously prescribed medications for COPD or other diseases.

The non-smoking study participants (non-smokers) were enrolled if they were >40 years of age, were healthy and not taking any medications, and had normal lung function according to the GOLD criteria for obstruction described above.

From 2007 to 2008, we collected baseline spirometric measurements and plasma samples from 345 participants. Follow-up spirometric measurements were taken 4 years later, from 2011 to 2012, and there were 295 participants with a baseline blood sample as well as baseline spirometry and follow-up spirometry. Post-bronchodilation values were used for the assessment of longitudinal change of lung function. The study was approved by the Ethics Committee of Lapland Central Hospital (4th June 2003 and 31st October 2006), and all participants provided written informed consent.

### Plasma samples

Peripheral whole venous blood was collected in EDTA tubes. Plasma was prepared by centrifugation for 10 to 15 min at 1500 rpm and stored at -80°C until analysis.

### Measurement of plasma sRAGE and HMGB1 concentrations

Plasma levels of sRAGE and HMGB1 were measured by commercially available ELISA kits (R & D Systems, Minneapolis, MN, USA and Uscn Life Science Inc, Wuhan, China, respectively) according to the manufacturer instructions. The detection limits for sRAGE and HMGB1 were 78 pg/mL and 0.238 ng/mL, respectively.

### Statistical analysis

The results are expressed as mean ± standard deviation (SD) if not stated otherwise. The analyses of variance (ANOVA) and t-test for independent groups were used to check for statistical significance in differences in participant characteristics and plasma levels of sRAGE and HMGB1 among the study groups. Spearman’s rank correlation was used to evaluate the associations of plasma sRAGE and HMGB1 concentrations with other variables. To estimate the independent effects of explanatory variables for the changes in lung functions during the 4 years, multivariate regression analysis was performed for each dependent lung function. The data were analysed with a statistical software package (SPSS for Windows, version 21.0; SPSS Inc; Chicago, IL).

## Results

### Participant characteristics

The baseline demographic and clinical characteristics of participants in each of the study groups (non-smokers, smokers without COPD, and smokers with COPD) are shown in Table 1. The smokers without COPD were significantly younger than the non-smokers and the smokers with COPD. There were no significant differences in BMI among the three groups. FEV_1_ was significantly lower in smokers with COPD than in the other two groups. FEV_1_/FVC was also significantly lower in smokers without COPD than in non-smokers (p = 0.014) and further reduced in smokers with COPD in comparison with that in smokers without COPD (p < 0.001).

**Table 1:** Baseline characteristics of study participants

	**All**	**Non-smokers**	**Smokers without COPD**	**Smokers with COPD**
Subjects, n	295	32	212	51
M/F	161/134	10/22	109/103	42/9
Age years	53.7 ± 9.3	56.0 ± 9.1	52.1 ± 8.8	58.9 ± 9.0
BMI	27.0 ± 3.8	27.0 ± 3.9	26.9 ± 3.8	27.3 ± 3.9
Smoking status				
Former smoker n (%)	56 (19%)	0	44 (21%)	12 (23%)
Current smoker n (%)	207 (70%)	0	168 (79%)	39 (77%)
Pack-years	26.8 ± 16.7	0	28.3 ± 14.1	37.6 ± 14.1
Symptom				
Only Cough n (%)	17 (5.9%)	0 (0%)	13 (6.1%)	4 (7.8%)
Only Sputum n (%)	58 (20.1%)	6 (23.1%)	47 (22.2%)	5 (9.8%)
Both Cough and sputum n (%)	97 (33.6%)	2 (7.7%)	71 (33.5%)	24 (47.1%)
Pre-bronchodilator				
FVC L	3.9 ± 0.9	3.7 ± 0.8	4.0 ± 0.9	3.9 ± 0.9
FVC % predicted	96.2 ± 12.5	102.6 ± 13.4	96.6 ± 11.5	90.9 ± 14.1
FEV1 L	3.0 ± 0.8	3.2 ± 0.7	3.2 ± 0.8	2.5 ± 0.7
FEV1 % predicted	90.8 ± 16.9	108.1 ± 13.5	94.6 ± 13.0	70.4 ± 15.5
FEV1/FVC %	76.8 ± 9.3	81.4 ± 5.3	79.5 ± 6.1	62.9 ± 9.4
Post-bronchodilator				
FVC L	4.0 ± 1.0	3.7 ± 0.8	4.0 ± 1.0	4.1 ± 1.0
FVC % predicted	96.7 ± 12.6	105.0 ± 9.9	96.5 ± 12.4	95.2 ± 13.4
FEV1 L	3.1 ± 0.8	3.1 ± 0.6	3.3 ± 0.8	2.6 ± 0.7
FEV1 % predicted	93.3 ± 16.3	110.6 ± 13.6	96.8 ± 12.8	74.3 ± 14.8
FEV1/FVC %	78.7 ± 9.3	84.3 ± 5.3	81.6 ± 5.5	62.9 ± 7.7

Values are mean ± SD unless stated otherwise.

*BMI*, Body mass index; *COPD*, Chronic obstructive pulmonary disease; *FEV*_*1*_, Forced expiratory volume in 1 s; *FVC*, Forced vital capacity.

### Baseline plasma sRAGE and HMGB1 concentrations

**Figure 1:**
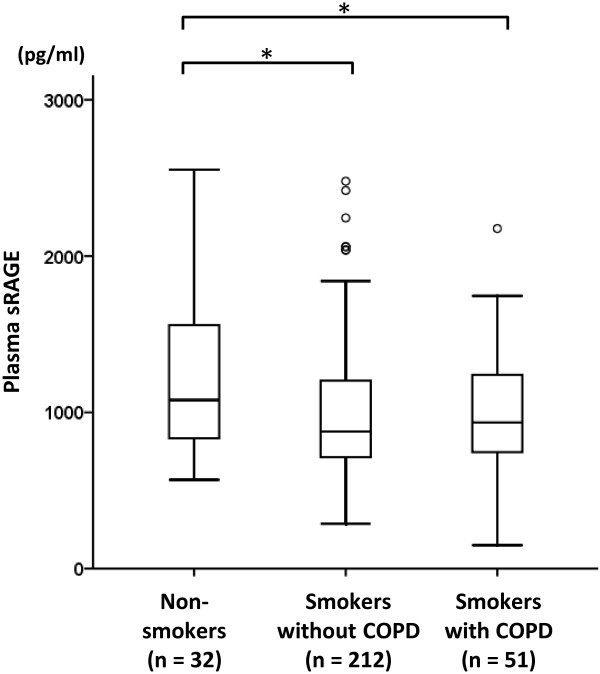
**Plasma levels of soluble receptor for advanced glycation end-products (sRAGE).** The boxes represent the 25th to 75th percentiles; the solid lines within the boxes show the median values; the whiskers are the 10th and 90th percentiles; and the points represent outliers. *: p < 0.05 and **: p < 0.01 (between 2 groups, t-test).

The baseline plasma levels of sRAGE (mean ± SD) were significantly lower in smokers without COPD and smokers with COPD (973.4 ± 426.5 pg/mL and 969.1 ± 406.0 pg/mL, respectively) than in non-smokers (1201.1 ± 483.6 pg/mL) (Figure 1). There was no significant difference in mean plasma HMGB1 concentrations among the three groups (non-smokers, 1.8 ± 1.1 ng/mL; smokers without COPD, 1.9 ± 1.1 ng/mL; smokers with COPD, 1.8 ± 1.7 ng/mL; figure not shown). There was no significant correlation between plasma sRAGE and HMGB1 levels in any of the subjects or in any of the groups in the present study (p > 0.05).

### Correlation of sRAGE and lung function

Plasma sRAGE levels were inversely correlated with BMI and pack-years and positively correlated with FVC and FEV_1_ (Table 2). In a subgroup analysis, there were weak but statistically significant correlations between plasma sRAGE concentrations and BMI and FVC and FEV_1_ only in smokers without COPD. There was a similar trend in smokers with COPD, but the p-value did not reach statistical significance (p > 0.05). There was no significant correlation between presence of chronic cough and/or sputum at the baseline visit and the plasma levels of sRAGE and HMGB1 in any of the subjects or among any of the study groups (p > 0.05). There was no significant association between plasma HMGB1 levels and baseline cross-sectional parameters in any of the groups (data not shown).

**Table 2:** Correlations between plasma sRAGE concentrations and baseline demographics

	**All**		**Non-smokers**		**Smokers without COPD**		**Smokers with COPD**	
	**(n = 295)**		**(n = 32)**		**(n = 212)**		**(n = 51)**	
	**r**	**p-value**	**r**	**p-value**	**r**	**p-value**	**r**	**p-value**
Age	-0.053	0.362	0.106	0.563	-0.110	0.109	-0.061	0.670
BMI	-0.139	0.017*	0.260	0.151	-0.162	0.018*	-0.272	0.053
Pack-year	-0.149	0.010*	-	-	-0.072	0.295	-0.262	0.063
FVC	0.130	0.026*	0.119	0.518	0.140	0.042*	0.131	0.360
FVC % predicted	0.190	0.001**	0.042	0.829	0.189	0.006**	0.163	0.252
FEV1	0.126	0.030*	0.116	0.526	0.133	0.054	0.151	0.291
FEV1 % predicted	0.136	0.020*	0.044	0.821	0.164	0.017*	0.176	0.217
FEV1/FVC	-0.029	0.620	0.070	0.245	-0.064	0.353	0.138	0.335

*: p < 0.05; **: p < 0.01 (correlation with plasma sRAGE levels by Spearman’s rank correlation coefficient).

*BMI*, Body mass index; *COPD*, Chronic obstructive pulmonary disease; *FEV*_*1*_, Forced expiratory volume in 1 s; *FVC*, Forced vital capacity.

### Correlation between reduced sRAGE and progression of airflow limitation

**Figure 2:**
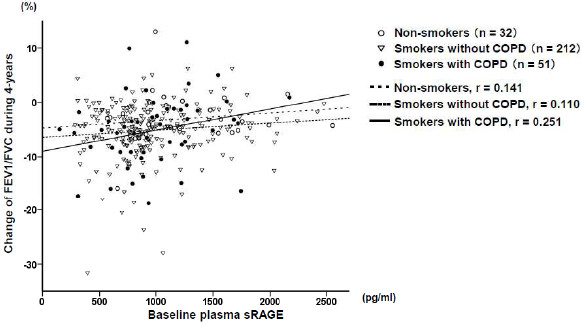
**Relationship between plasma sRAGE levels at baseline and the change of FEV**_**1**_**/FVC during a 4 year follow-up.** sRAGE: soluble receptor for advanced glycation end-products.

Longitudinal changes of FEV1 (mean±SEM) in non-smokers, smokers without COPD, and smokers with COPD during the 4-year study period were -243 ± 37 mL, -325 ± 18 mL, and -355 ± 41 mL, respectively. The lower plasma sRAGE concentrations in all groups were significantly associated with longitudinal decline of FEV_1_/FVC (ΔFEV_1_/FVC) (Table 3, Figure 2), while there was no significant association between longitudinal changes of lung function and demographic characteristics or plasma HMGB1 levels (data not shown). Additionally, a subgroup analysis showed that a lower sRAGE concentration was significantly associated with a greater decline of FEV_1_/FVC in smokers with COPD (Table 3, Figure 2). For smokers with COPD, a trend became evident between a decreased plasma sRAGE level and a longitudinal decline in FEV_1_ (Table 3, Additional file 1: Figure S1). There were 13 smokers with COPD who had started an inhaled medication, i.e. a bronchodilator and/or corticosteroids, at the follow-up visit. In the remaining 38 smokers with COPD, there was still a significant correlation between baseline sRAGE levels and decline of FEV_1_/FVC (r = 0.362, p = 0.025). Finally, multiple stepwise regression analysis was performed in order to investigate the association of FEV_1_/FVC decline with sRAGE levels together with previously reported predictors of lung function decline, including age, sex, BMI, current smoking status, and baseline lung function [20-22]. In this multivariate analysis, lower baseline sRAGE levels were an independent predictor for a more precipitous decline of FEV_1_/FVC even after adjusting for all other variables (p = 0.019) (Table 4). Age (p < 0.001), baseline FEV_1_/FVC (p = 0.013), and current smoking (p = 0.014) were also independent predictors for ΔFEV_1_/FVC.

**Table 3:** Correlations between plasma sRAGE concentrations and longitudinal changes in lung function

	**All**		**Non-smokers**		**Smokers without COPD**		**Smokers with COPD**	
	**(n = 295)**		**(n = 32)**		**(n = 212)**		**(n = 51)**	
	**r**	**p-value**	**r**	**p-value**	**r**	**p-value**	**r**	**p-value**
ΔFVC	-0.043	0.466	-0.142	0.438	-0.033	0.637	-0.031	0.828
ΔFVC % predicted	-0.047	0.432	-0.187	0.540	-0.033	0.632	-0.014	0.922
ΔFEV1	0.061	0.294	-0.115	0.529	0.008	0.907	0.240	0.090
ΔFEV1 % predicted	0.059	0.332	-0.215	0.481	0.029	0.680	0.269	0.056
ΔFEV1/FVC	0.149	0.010*	0.259	0.153	0.053	0.445	0.326	0.019*

*: p < 0.05; (correlation with plasma sRAGE levels, by Spearman’s rank correlation coefficient).

*COPD*, Chronic obstructive pulmonary disease; Δ: changes from baseline after 4 years; *FEV*_*1*_, Forced expiratory volume in 1 s; *FVC*, Forced vital capacity; *sRAGE*, Soluble receptor for advanced glycation end-products.

**Table 4:** **Multivariate stepwise analysis of all participants with ΔFEV**_**1**_/FVC as the dependent variables*

	**β**	**t test**	**p value**
Age	-0.258	-4.295	<0.001
Baseline FEV1/FVC	-0.160	-2.494	0.013
Current smoking	-0.140	-2.466	0.014
Plasma sRAGE	0.134	2.364	0.019

Δ: changes from baseline after 4 years; *FEV*_*1*_, Forced expiratory volume in 1 s; *FVC*, Forced vital capacity; *sRAGE*, Soluble receptor for advanced glycation end-products.

*(n = 295). The other independent variables included in the model were sex and BMI.

## Discussion

In the present study, we evaluated plasma concentrations of sRAGE and HMGB1 in non-smokers, smokers without COPD, and smokers with COPD, and we subsequently examined the association between these two markers using baseline demographic data and the longitudinal decline of lung function during a 4-year follow-up period. Baseline plasma sRAGE levels were significantly lower in smokers with and without COPD than in non-smokers. Moreover, plasma sRAGE concentrations were significantly associated with longitudinal declines of FEV_1_/FVC, and this association remained significant even after controlling for demographics and baseline lung function. In contrast, there was no significant difference in plasma HMGB1 levels among the three groups, nor were there any significant associations among plasma HMGB1 concentration, baseline lung function, and decline in lung function during the follow-up period in any group.

To the best of our knowledge, this is the first study to demonstrate that reduced plasma sRAGE levels are associated with progression of airflow limitation. Interestingly, this association was apparent in smokers with COPD, but we could not perform a subgroup multiple regression analysis for the COPD group because of its limited sample size. Previously, circulating sRAGE in patients with COPD has been associated with emphysema severity, impaired diffusion capacity, and airway neutrophilic inflammation [5-8], supporting a possible role of RAGE in alveolar integrity and the anti-inflammatory properties of sRAGE [23-25]. A recent longitudinal study found significant associations between circulatory sRAGE levels and decline of lung density in CT scans in patients with moderate to severe COPD, suggesting the possible association between RAGE and disease progression [26]. The present study showed that lower plasma sRAGE levels were predictive for progression of airflow limitation, which further supports the theory that RAGE might have a protective role against progression of COPD. Further investigation is needed to clarify the mechanism of this association.

In the present study, the differences in plasma sRAGE concentrations between smokers with COPD and smokers in whom post-bronchodilator spirometry results did not meet the COPD criteria were not significant. It should be noted that most of the participants in the COPD group had only mild to moderate airflow limitation in the present study. Recent cross-sectional studies have shown that plasma sRAGE levels are decreased especially in COPD patients who have severe disease [4,8,9], and circulatory sRAGE levels were significantly reduced in accordance with advanced GOLD stage in the COPD patients of the ECLIPSE cohort [8]. On the other hand, Boschetto et al. did not find a significant difference in plasma sRAGE levels between healthy volunteers and COPD patients with mild to moderate airflow limitation, which is in agreement with the findings of the present study [14]. Therefore, although we did not observe a significant difference in circulatory levels of sRAGE between smokers with COPD and smokers without COPD, our results could still indicate that an sRAGE deficiency might be associated with more advanced COPD. A future large-scale study will be warranted to determine whether plasma sRAGE would be useful as an early diagnostic marker for COPD. Furthermore, we believe that the assessment of emphysema by CT scans or diffusion capacity should be further investigated, because these parameters seem to be significantly associated with sRAGE levels independently of airflow limitation in patients with COPD [6-8].

We found no difference between plasma HMGB1 levels among non-smokers, smokers without COPD and smokers with COPD, and there was no significant association between plasma HMGB1 levels and spirometric measurements. These findings varied from those of the recent reports that indicated plasma HMGB1 levels were elevated in patients with COPD, especially in those with severe airflow limitation [13]. Hou et al. found significantly elevated plasma HMGB1 levels using ELISA in COPD patients in comparison with normal controls [13]. We used the same ELISA method in our study, and the HMGB1 levels in the controls were comparable with those in the previous study [13]. However, a striking difference is that Hou et al. included COPD patients with severe airflow limitation (mean %FEV_1_, 34.99%), and the HMGB1 levels of those COPD patients were higher than those of the control group. Shang et al. measured serum HMGB1 levels by western blot in patients with non-small cell lung cancer and in patients with COPD [27]. In their study, the serum HMGB1 levels were higher in COPD patients with more severe airflow limitation (mean %FEV_1_, 49%) when compared with the results in the present study, and they found that patients with lung cancer had even higher levels of serum HMGB1. In another recent study that has reported elevated plasma HMGB1 levels in patients with mild to moderate COPD, 82% of the COPD patients had comorbid lung cancer [15]. In fact, our study is the first to compare circulatory HMGB1 levels between never-smokers, a control group of smokers without COPD, and smokers with early stage COPD and no comorbidities, and the inconsistencies of the HMGB1 levels in the COPD patients in the present study and those of the previous studies are probably related to the differences in the background characteristics of the study participants.

There were several limitations in the present study. First, the number of participants in the non-smoker and smoker with COPD groups was relatively small. A larger sample size for the COPD group would be necessary to confirm the association between sRAGE and decline of lung function in a subgroup analysis. Additionally, because there were only 9 female participants in the COPD group, we could not analyse gender-related differences in longitudinal decline of lung function in the COPD group. Secondly, we did not perform high-resolution computed tomography or diffusion capacity studies. Thirdly, the follow-up period of 4 years was relatively short, but this was due to the study design of a longitudinal analysis among apparently healthy middle-aged to elderly populations. On the other hand, this study had significant strengths. None of the participants had any other exposures and no participants in any of the groups, including those chronic smokers who were diagnosed with COPD, had any comorbidities and were not taking any medications at the time of enrolment [16].

## Conclusion

In conclusion, we have demonstrated that decreased plasma sRAGE levels are associated with the progression of airflow limitation, especially in smokers with COPD. This suggests that RAGE might have a protective role against the progression of smoking-induced lung damage, although further studies are warranted to confirm the present observation and to clarify the relationships between the RAGE/HMGB1 pathway and its role in the pathophysiology of COPD.

## Abbreviations

COPD: Chronic obstructive pulmonary disease; FEV1: Forced expiratory volume in one second; FVC: Forced vital capacity; HMGB1: High mobility group box 1; sRAGE: Soluble receptor for advanced glycation end-products.

## Competing interests

The authors declare that they have no competing interests.

## Authors’ contributions

HI participated in the design of the study, performed part of the statistical analysis and drafted the manuscript. JG carried out the ELISA measurements and participated in the preparation of the manuscript. VP contributed in the interpretation of the results and in the writing process. TT participated in the recruitment and interview of the subjects and their characterization and was responsible for the lung function analyses. PN contributed to the statistical analyses and interpretation of data. WM conceived the study, and participated in its design and coordination, and helped to draft the manuscript. All authors have read and approved the final manuscript.

## Pre-publication history

The pre-publication history for this paper can be accessed here:

http://www.biomedcentral.com/1471-2466/14/68/prepub

## Supplementary Material

Additional file 1: Figure S1 Relationship between plasma sRAGE levels at baseline and the change of FEV_1_ during a 4 year follow-up. sRAGE: soluble receptor for advanced glycation end-products. Click here for file
