# Supplementary figures and images for: Soluble receptor for advanced glycation end-products and progression of airway disease
Source: BMC Pulm Med. 2014 Apr 24;14:68. doi: 10.1186/1471-2466-14-68 (PMC4021457; doi:10.1186/1471-2466-14-68)

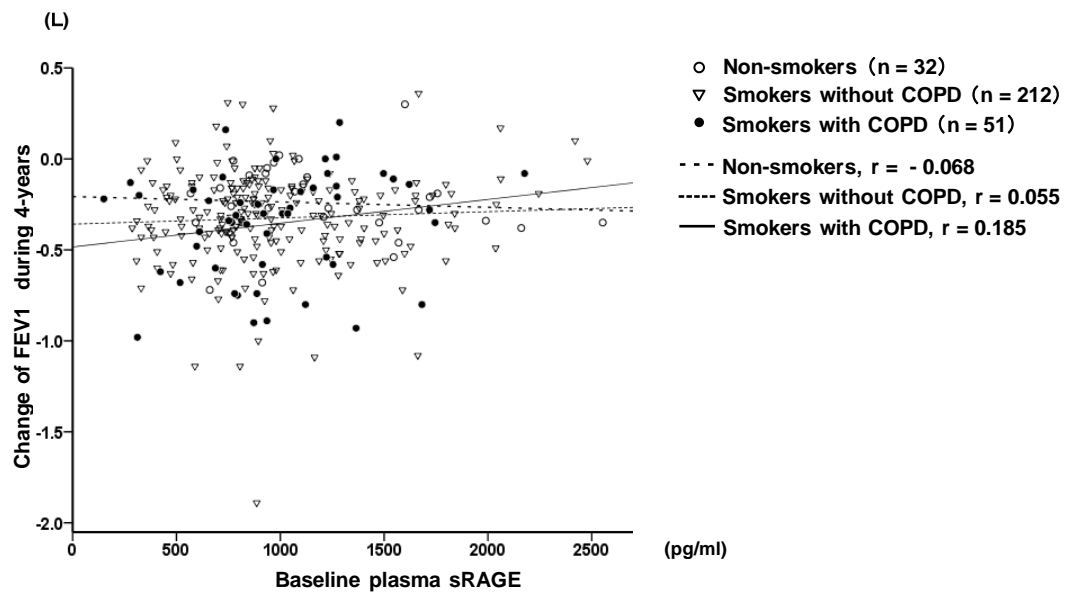

Supplement: Additional file 1: Figure S1 — Relationship between plasma sRAGE levels at baseline and the change of FEV1 during a 4 year follow-up. sRAGE: soluble receptor for advanced glycation end-products. [file 1471-2466-14-68-S1.pdf]
